# Revisiting doxycycline in pregnancy and early childhood – time to rebuild its reputation?

**DOI:** 10.1517/14740338.2016.1133584

**Published:** 2016-01-25

**Authors:** Ruby Cross, Clare Ling, Nicholas P. J. Day, Rose McGready, Daniel H. Paris

**Affiliations:** ^a^ Shoklo Malaria Research Unit, Mahidol-Oxford Tropical Medicine Research Unit, Faculty of Tropical Medicine, Mahidol University, Mae Sot, Tak, Thailand; ^b^ Mahidol-Oxford Tropical Medicine Research Unit (MORU), Faculty of Tropical Medicine, Mahidol University, Bangkok, Thailand; ^c^ Centre for Tropical Medicine & Global Health, Nuffield Department of Clinical Medicine, University of Oxford, Oxford, UK

**Keywords:** Doxycycline, tetracycline, pregnancy, rickettsiosis, prenatal exposure, side effects, teratogenicity, major congenital anomalies, tooth discolouration, *Orientia tsutsugamushi*, *Rickettsia typhi*, scrub typhus, murine typhus, undifferentiated fever

## Abstract

***Introduction:*** Doxycycline is highly effective, inexpensive with a broad therapeutic spectrum and exceptional bioavailability. However these benefits have been overshadowed by its classification alongside the tetracyclines – class D drugs, contraindicated in pregnancy and in children under 8 years of age. Doxycycline-treatable diseases are emerging as leading causes of undifferentiated febrile illness in Southeast Asia. For example scrub typhus and murine typhus have an unusually severe impact on pregnancy outcomes, and current mortality rates for scrub typhus reach 12-13% in India and Thailand. The emerging evidence for these important doxycycline-treatable diseases prompted us to revisit doxycycline usage in pregnancy and childhood.

***Areas Covered:*** A systematic review of the available literature on doxycycline use in pregnant women and children revealed a safety profile of doxycycline that differed significantly from that of tetracycline; no correlation between the use of doxycycline and teratogenic effects during pregnancy or dental staining in children was found.

***Expert Opinion:*** The change of the US FDA pregnancy classification scheme to an evidence-based approach will enable adequate evaluation of doxycycline in common tropical illnesses and in vulnerable populations in clinical treatment trials, dosage-optimization pharmacokinetic studies and for the empirical treatment of undifferentiated febrile illnesses, especially in pregnant women and children.

## 1. Introduction

Doxycycline is a highly effective but underappreciated antimicrobial, with a broad therapeutic spectrum, exceptional bioavailability and very little evidence of serious adverse events (SAEs). Doxycycline is cheap and the most popular tetracycline derivative currently available (Table 1). Doxycycline is used to treat infections of high global impact including malaria (prevention and as a partner drug for treatment), sexually transmitted infections (STI, i.e. pelvic inflammatory disease, chlamydia, syphilis), rickettsial illnesses (leading causes of treatable undifferentiated febrile illness in Asia), Lyme disease, skin infections and acne. It is efficacious for treating bio-threat and outbreak-associated infections, like tularemia, cholera and bubonic plague, as well as methicillin resistant *Staphylococcus aureus* (MRSA).[1] Unfortunately, doxycycline was developed after the tetracyclines had been labeled as potentially harmful because of severe adverse effects, including teratogenicity, permanent yellowish-brown teeth discoloration after *in utero* exposure and in children under 8 years of age and more rarely fatal hepatotoxicity reported in pregnant women.[2–12] This ‘Tetracycline Class Effect’ led to the classification of doxycycline as a category D drug by the USA Food and Drug Administration (FDA) Pregnancy Category classifications based on A, B, C, D, X categories.[13,14] Consequently its use has been limited, especially in two vulnerable patient groups: pregnant women and children. Although this classification system has recently been replaced with an evidence-based approach, the consequences of the previous labeling of doxycycline as a class D drug are that very limited data of human clinical trials in pregnancy and childhood are available, as are pharmacokinetic studies addressing dosage regimen optimization and drug efficacy evaluation.

**Table 1.** Current treatment recommendations in pregnant and non-pregnant patients when doxycycline is ‘drug of choice’.

Infectious disease	Causative organism	Adult non-pregnant	Pregnant women
Rocky mountain spotted fever (RMSF)	*Rickettsia rickettsii*	Orally or i.v. doxycycline is the DOC for the treatment of RMSF in both adults and children, except for pregnant women. A dose of 100 mg for 7 days or for 2 days after temperature has subsided. Some recommend an initial one time loading dose of 200 mg.[15–17]	Chloramphenicol is preferred: 50 mg/kg per day in four divided doses p.o. for 7 days.[15]
Genito-urinary and non-genitourinary disease^a^	*Mycoplasma* spp. *Ureaplasma urealyticum*	*M. hominis*, unlike other *Mycoplasma* and *Ureaplasma* spp., is not susceptible to macrolides, use doxycycline 100 mg IV/p.o. b.i.d. x 7–10 days.[18–21] *M. pneumoniae*: Doxycycline 100 mg IV/p.o. b.i.d. x 7–10 days.[21] *Ureaplasma* spp. are typically susceptible *in vitro* to the tetracyclines e.g. doxycycline.[19] Tetracycline resistant *M. hominis* and *ureaplasma* spp. reported.	*M. hominis*: preferably clindamycin.[22] *U. urealyticum* clarithromycin or moxifloxacin.[22,23] *M. genitalium*: azithromycin.[23] *M. pneumoniae*: azithromycin 500 mg p.o. day one and then 250 mg p.o. x 4 days or levofloxacin 750 mg p.o./IV x 5 days.[21]
Human ehrlichiosis (HME) and anaplasmosis (HGA)	*Ehrlichia chaffeensis*, *Anaplasma phagocytophilum*	Recommendations are based on clinical case series and *in vitro* data. Both tetracycline (mostly doxycycline) and chloramphenicol appear to be effective clinically.[24] *E. chaffeensis* is susceptible only to doxycycline.[25]	No guidelines exist for the treatment of ehrlichiosis in pregnancy. Experts believe that doxycycline is warranted in the treatment of life-threatening HME or HGA during pregnancy.[26] Doxycycline has been used successfully and safely in a few cases in pregnant women with HME or HGA.[27,28] Rifampin has been effective in small numbers of pregnant women with HGA.[29]
Chlamydia trachomatis infection	*Chlamydia trachomatis*	CDC recommends: 1 g azithromycin orally as single dose, or 100 mg doxycycline orally (b.d.) for 7 days for uncomplicated genito-urinary infection. Alternate regimens include erythromycin 500 mg orally four times a day (q.i.d.) or ofloxacin 300 mg orally (b.d.) for 7 days.[30,31]	Azithromycin 1 g orally given as a single dose or amoxicillin 500 mg orally three times daily for 7 days or erythromycin 500 mg orally four times a day (q.i.d.) if either of these is not tolerated.
Brucellosis	*Brucella* spp. *B. melitensis, B. abortus, B. suis*, and *B. canis*	Doxycycline plus aminoglycoside.[32] Adults and children aged >8 are recommended 100 mg doxycycline p.o. bid x 6 weeks and gentamicin 5 mg/kg IV once daily x 7 days or rifampin 600–900 mg p.o. once daily x 6 weeks to replace gentamicin.[33]	World Health Organization (WHO) calls for the use of rifampicin monotherapy.[34] Sanford guide suggests trimethoprim-sulfamethoxazole (TMP-SMX). 5 mg/kg of TMP component p.o. b.i.d. plus Rifampin 600–900 mg p.o. q24 h for 4 weeks.[33]
Lyme disease	Borrelia species: *B. burgdorferi* (in US) and *B. afzelii, B. garinii* (Europe, Asia)	Equal efficacy: doxycycline, amoxicillin, cefuroxime axetil early Lyme disease. Doxycycline is drug of choice as it treats a potential coinfecting agent, *Anaplasma phagocytophilum*. Of the oral agents commonly used to treat Lyme disease, doxycycline has the best penetration into the central nervous system.	Early Lyme disease 500 mg of amoxicillin three times a day for 14–21 days.[35–37]
Anthrax	Individuals exposed to aerosolised *Bacillus anthracis*	Ciprofloxacin 500 mg every 12 h in adults or doxycycline 100 mg every 12 h in adults. Recommended PEP duration is 60 days; coverage of incubation period and protection until sufficient vaccine-induced immunity.[38] Treatment with doxycycline + clindamycin, or azithromycin + rifampicin, according to guidelines.	Ciprofloxacin 500 mg orally every 12 h is considered the first-line drug for PEP, or clindamycin 600 mg every 8 h or doxycycyline 100 mg every 12 h.[13]
Q fever	*Coxiella burnetii*	Doxycycline 100 mg p.o. b.i.d. for 2–3 weeks.[39]	TMP-SMX (Trimethoprim-sulphamethoxazole) DS Table 1 p.o. b.i.d. for duration of pregnancy to prevent premature labour.[39]
Cholera	*Vibrio cholerae*	Doxycycline 300 mg p.o. as a single dose (plus rehydration solution).[40]	Azithromycin 1 gm p.o. single dose.[40]
Malaria	*Plasmodium falciparum, Plasmodium vivax*	Treatment: artesunate followed by doxycycline 100 mg po twice daily/ seven days or clindamycin 20 mg base/kg/day orally divided three times daily for 7 days.[41] Prevention: doxycycline.[42]	For uncomplicated *P. falciparum*: artemether-lumefantrine 4 tablets (80 mg/480 mg) single dose, then 4 tablets after 8 h and then after every 12 h for 2 days.[43] or Quinine sulphate 10 mg/kg p.o.t. i.d. x3 days (7 days SE Asia) + clindamycin 20 mg/kg/day divided b.i.d. x 7 days.[43] Prophylaxis for chloroquine-resistant malaria: mefloquine 250 mg 1 tablet p.o. once weekly, 2–3 weeks before, during and 4 weeks after travel to at-risk area.[42,44]

Tetracyclines, contraindicated as risk of fetal bone and teeth malformations and hepatotoxicity in the mother except: (a) third trimester because of ‘Gray syndrome’ in premature infants and newborns; (b) life threatening situations with multiorgan failure, as doxycycline is thought to be superior to chloramphenicol and protecting the life of the mother is of primary importance; (c) when chloramphenicol cannot be obtained. The risks and benefits of the choice of antibiotic must be discussed with the patient and determined on a case by case basis. If used, doxycycline is the preferred tetracycline.[26,45] ^a^Infections that have been linked to *M. hominis* include: Pelvic inflammatory disease (PID), chorioamnionitis, postpartum and postabortal fever, pyelonephritis, central nervous system infections, septicemia, wound infections, joint infections, upper and lower respiratory tract infections, endocarditis, neonatal bacteremia and meningitis, neonatal abscesses. Ureaplasma spp. have been linked to: chorioamnionitis, postpartum and post-abortal fever, congenital pneumonia, neonatal bacteremia, neonatal abscesses.

In this systematic review we aimed to critically summarize the published literature on the therapeutic benefits of doxycycline, delineate the reasons for its limited usage in these vulnerable populations and identify clinically relevant knowledge gaps for the evaluation of doxycycline in pregnancy and childhood.

## 2. Methods

### 2.1. Information sources, search strategy and study selection

Articles were identified through electronic resources by scanning library index catalogs and reference lists of relevant articles. Searches were performed using PubMed, SciELO (Scientific Electronic Library Online), Research Gate, Google Scholar and Google. These electronic databases were searched using ‘doxycycline’ alone or in combination with additional words such as ‘hepatotoxicity’ OR ‘pregnancy’ OR ‘children’ OR ‘teeth’ OR ‘side-effects’, and results were reviewed manually. All articles in English were reviewed and duplicate search results were removed manually. RC and CL acted as primary reviewers and RMG and DHP as secondary reviewers. Abstracts and titles from all search results were assessed for inclusion in the review and the full article was obtained if relevance was unclear. All reports/papers selected for retrieval were assessed by two independent reviewers for methodological validity prior to inclusion in the review and followed a protocol based on the PRISMA statements for systematic reviews (suppl. file S1 Checklist). Any disagreements between the reviewers were resolved through discussion with the secondary reviewers or with an independent assessor (NPD).

**Figure 1.**
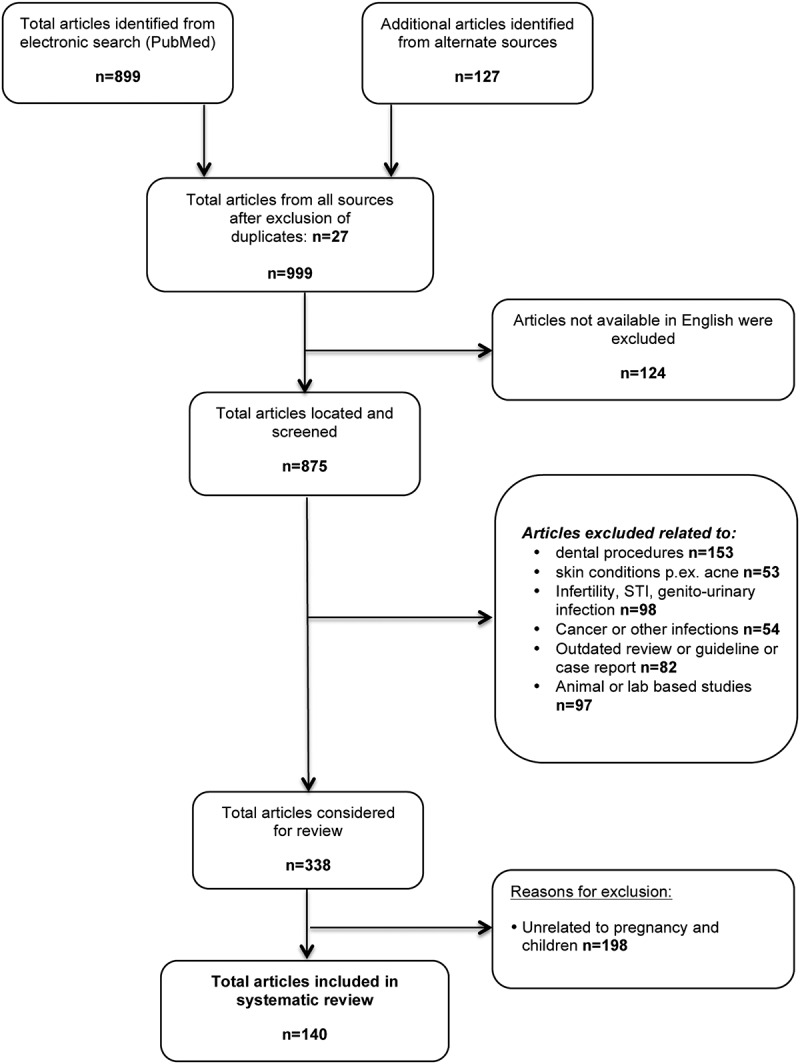
Studies selection process flow chart for this systematic review.

Authors were not contacted regarding further information, as many publications were not recent. Unpublished literature was not included and abstracts were not considered if a full article was not obtainable (Figure 1). The primary outcomes of the analysis related to all relevant adverse effects for tetracycline versus doxycycline in the literature and included as primary outcomes: irreversible tooth-staining, bone growth inhibition and teratogenicity; whereas secondary outcome measures included hepatotoxicity and nephrotoxicity relating to doxycycline. This systematic review was not registered. Statistical analysis used, the two sample z-test for comparisons of proportions to show differences between treatment groups, exposure status and adverse events (AEs) where amenable, and p-values and risk differences (95% CI) reported, using Stata version 14.0, Stata Corp., College Texas, USA.

## 3. Results

Overall the collated evidence was limited by the lack of high quality manuscripts, the age of available publications and the lack of recent evidence. The selection procedures led to inclusion of 140 articles, but also exclusion of a large number of reports, mainly relating to treatment of dental procedures (Figure 1 flow chart). The restricted amount of available data amenable to statistical meta-analysis reflects the reputation of doxycycline and provides indirect supportive evidence towards the objectives of this review (Table 2). Further, the difficulties of assessing the objectivity of outcomes, like tooth staining, which can only be assessed after a long follow-up, represented limitations for the statistical pooling of results.

**Table 2.** Summary and comparison of reported adverse effects for tetracycline versus doxycycline.

	Tetracycline		Doxycycline		Difference in proportion		
Adverse effect	Exposure with AE	Exposure without AE	Exposure with AE	Exposure without AE	p-value (RD; 95% CI)	Comments	References
Irreversible tooth-staining	928	313	1	38	p < 0.0001 (0.72; 0.67–0.78)	Doxycycline given to premature infants aged 4–55 days, 2 mg/kg for 6–17 days. 1/25 with slight spotted discoloration of upper incisors.	[3,6,9,46–60]
Reversible bone growth inhibition	25	0	NA	NA	*^#^*	Mean fibula growth inhibition of up to 40% length during tetracycline treatment, rapid compensatory growth rate and return to normal upon discontinuation of drug.	[61]
Teratogenicity	16	214	56	1949	p = 0.001 (0.04; 0.07–0.75)	Doxycycline: no evidence for increased teratogenicity observed, OR was equal to or smaller than controls. Oxytetracycline with higher teratogenic risk: OR (95% CI): neural tube defects (9.7, 2.0–47.1), cleft palate (17.2, 3.5–83.5), MCAs (12.9, 3.8–44.3).	[62–65]
Hepatotoxicity	77*	0	0	62**	*^$^*	Doxycycline recipients and matched controls had similar incidences of hepatotoxicity compared to tetracycline in 3377 cases. *Tetracycline: Current use OR (95% CI) 3.7, 1.19–11.45 Past use OR (95% CI) 2.72, 1.26–5.85 **Doxycycline: Current use OR (95% CI) 1.49, 0.61–3.62 Past use OR (95% CI) 1.74, 0.99–3.06	[2,4,5,7,8,10–12]
Nephrotoxicity	7	0	0	0	*^$^*	All patients had concomitant liver involvement.	[10,66]
Total SAEs	1053	527	57	2049		No doxycycline-associated SAEs in n = 2049, except for one case with slight discoloration of upper incisors.	

AE = adverse effect; RD = risk difference; OR = odds ratio; MCA = major congenital anomaly; NA = not available; # comparison not possible; $ comparison not valid (studies not congruent); data of patients with hepatotoxicity under tetracycline* or doxycycline** use, respectively. Statistically increased risk of hepatotoxicity was described with tetracycline, but not doxycycline (current use OR 3.70, 95% CI 1.19–11.45; past use OR 2.72, 95% CI 1.26–5.85), ref [67]. Comparisons of proportions were calculated for ‘Irreversible tooth-staining’ and ‘Teratogenicity’ only, as studies were not congruent for other endpoints.

### 3.1. Effectiveness for infection treatment

The first tetracycline antibiotics discovered were Aureomycin in 1948, oxytetracycline in 1949 and tetracycline in 1953.[68] Tetracyclines are bacteriostatic drugs that inhibit microbial protein synthesis by reversible binding to the 30S subunit of the bacterial ribosome.[69] Initially tetracycline was used as a broad-spectrum antibiotic with activity against Gram-positive and Gram-negative bacteria including aerobes and anaerobes. Although some bacteria have developed resistance to tetracycline, it remains highly effective against intracellular pathogens, such as *Chlamydia*, *Rickettsia* and *Mycoplasma* species. Following its use, it became apparent that tetracycline adversely affects calcium orthophosphate metabolism in growing bones and teeth.[3,6,61,70] Subsequently, synthetic modifications to the basic four-ring structure of tetracycline were made as an attempt to improve efficacy and reduce adverse effects, resulting in drugs such as oxytetracycline, chlortetracycline and doxycycline.

Doxycycline was immediately popular after its FDA approval in 1967 because of the simplified once (or twice)-a-day dosage regimen, rather than the four times daily dosing scheme for tetracycline. Minocycline, the semi-synthetic sister drug, targets a similar antimicrobial spectrum, is more lipophilic than doxycycline and achieves higher concentrations in the CNS and skin.[71,72] Both drugs attain comparable serum levels, but unfortunately the higher cerebral spinal fluid and CNS concentrations of minocycline are associated with dose-limiting vestibular side effects. Although the incidence of AEs with each of these two drugs is very low, doxycycline is associated with fewer reported AEs.[72] Doxycycline is prescribed more often and remains the preferred antibiotic for treatment of early Lyme borreliosis, as well as being the first line regimen for Rocky Mountain spotted fever (RMSF), scrub typhus, murine typhus, leptospirosis, human granulocytic ehrlichiosis (HGE) and anthrax (in combination with rifampicin or clindamycin).[14,73–75] Doxycycline was FDA approved for use in pregnant women following exposure to biothreat agents, including *Bacillus anthracis, Yersinia pestis* and *Francisella tularensis*, despite being categorized as a Class D agent.[13,14]

Doxycycline is available as Doxy-hyclate (hydrochloride) and Doxy-monohydrate salts, although the salt compositions do not affect the efficacy of doxycycline, their absorption is negatively influenced by substances containing aluminum, calcium, iron, magnesium, zinc or bismuth subsalicylate if these are consumed within 2–3 h before or after doxycycline intake (i.e. antacids, dairy products etc.).

### 3.2. Potential for adverse teratogenic effects

Tetracyclines are on the list of proven human teratogens and therefore are contraindicated in pregnancy.[76] Tetracyclines and derivatives like oxytetracycline, present a documented teratogenic risk to the fetus, especially during the second trimester of pregnancy. They are associated with higher rates of neural-tube defects, cleft palate and other major congenital abnormalities (MCAs).[62] These adverse effects have led to the contraindication of tetracyclines in pregnancy, lactation and for children under 12 years of age. Because of the ‘tetracycline class effect’ these associations were applied to doxycycline and stated on the data sheet by Pfizer without citing any evidence of teratogenicity.[77]

There is no evidence in the current literature of any human teratogenicity following the use of doxycycline during pregnancy.[63] Cumulative evidence suggests that the use of doxycycline during the first trimester is not associated with any increased risk to the growing fetus.[64,65,78] All published reports on doxycycline-exposed pregnancies with mother and infant follow up data (AE reported in n = 46, no AE reported in n = 1949; total n = 2005) are summarized in Table 2.[62–65]

A large retrospective study based on the Hungarian Case–Control Surveillance of MCAs included 51,319 babies (32,804 healthy control infants and 18,515 cases with MCAs) born between 1980 and 1992, and evaluated women who took doxycycline at some point during pregnancy (Table 3).[64] There were 63/32,804 (0.19%) healthy control infants whose mothers had received doxycycline treatment during pregnancy, compared to 56/18,515 (0.30%) in the MCA cases, resulting in an odds ratio (OR) of 1.6 (95% CI 1.1–2.3) for major MCAs in women receiving doxycycline at any time during pregnancy. The authors raise the issue of small sample size (12 cases vs. 13 controls for the second and third months) and the case–control analysis did not show teratogenic potential of doxycycline in the critical period of any congenital-abnormality group.[64] Czeizel *et al*. concluded on the basis of these large case–control studies that doxycycline presents very little, if any, teratogenic risk to the fetus, stating that: ‘ *… the teratogenic risk of [certain] drugs in humans is exaggerated and it has several unfortunate consequences: negligence in necessary drug use, unnecessary anxiety in pregnant women, and termination of planned pregnancies without any reasonable cause …’*.[62,79]

**Table 3.** A comparison of antibiotics reviewed in the Hungarian Congenital Anomaly Registry.

Antibiotic taken during pregnancy (all trimesters)	Offspring with MCAs	Offspring with no MCAs	Statistical analysis OR (95% CI)	References
Doxycycline	56/18,515 (0.3%)	63/32,804 (0.19%)	1.6 (1.1–2.3)	[64]
Erythromycin	113/22,865 (0.5%)	172/38,151 (0.5%)	1.1 (0.9–1.4)	[80]
Chloramphenicol	52/22,865 (0.23%)	51/38,151 (0.13%)	1.7 (1.2–2.5)	[81]
Ampicillin	1643/22,865 (7.2%)	2632/38,151 (6.9%)	1.0 (0.7–1.2)	[82]
Oxytetracycline	216/22,865 (0.9%)	214/38,151 (0.6%)	1.7 (1.4–2.1)	[62]

The Hungarian Congenital Anomaly Registry was established in 1980 and has the largest case–control data set of major congenital anomalies (MCA) in the world. The total number of participants enrolled was at least 51,319 pregnant women. Of all 18,515 women who had an infant with a MCA, 0.3% had received doxycycline. Of the 32,804 women who had offspring with no MCA, 0.19% had inadvertently taken doxycycline (the majority during the first and second months of gestation 44.5%). Case–control pair analysis showed no significant increase in doxycycline treatment in second and third months of pregnancy in any group of MCA. MCA: major congenital anomaly; OR: odds ratio.

Cooper *et al*. performed a large retrospective study of 30,049 infants born between 1985 and 2000 recorded in the Tennessee Medicaid Database. The overall congenital malformation rate in new-borns exposed *in utero* to antibiotic drugs recommended for bioterrorism-related scenarios was 2.9% (ranging from 2.5 to 3%). The infants born to women who had received doxycycline during pregnancy (n = 1843), had a MCA rate of 2.5%, which was actually lower than the average MCA background rate for all exposed infants (2.9%) – median treatment duration was 9.6 days; 200 mg oral per day.[63] Multivariable analysis provided no evidence of greater risk for malformations in infants with fetal exposure to doxycycline than unexposed infants during the first 4 months of pregnancy (n = 1691, RR: 0.85, 95% CI: 0.59–1.23) or anytime during pregnancy (RR: 0.84, 95%: CI 0.59–1.19). Although the study did not record the gestational stage at which the mothers received doxycycline, 42 of 46 MCAs (91.3%) occurred in the first 4 months of gestation, suggesting that most women, unaware of their pregnancy, received treatment in the first trimester. The authors concluded that there was no increased risk of MGAs after exposure to doxycycline during pregnancy.[63]

A study investigating mycoplasma infections in 81 pregnancies identified 43/81 (53%) cases that received doxycycline for 10 days during the first trimester and reported that all infants were normal at 1 year of age; this however in a sample size too small to safely conclude that the drug was ‘safe’ and ‘free of adverse effects’.[65] One review on the prophylaxis and treatment of anthrax in pregnant women found no clear association between exposure to doxycycline and MCAs.[13] The overall available evidence suggests that the potential for any teratogenic effect in pregnant women taking doxycycline is minimal (Table 2).[14,64,78,83] Multiple studies conducted in different animal species between 1967 and 1980 found no teratogenic effects of doxycycline in pregnancy. Mouse and rabbit studies showed that there was no increase in congenital anomalies in animals treated with up to six times the maximal human therapeutic dose (3.33 mg/kg), but an increase of skeletal anomalies and decrease of fetal weight were observed at doses >17 times the human therapeutic dose.[84,85] No teratogenicity was observed at up to 100 times the maximum therapeutic dose in rats and monkeys.[84,86] It was concluded that an increased risk of teratogenicity is ‘*unlikely*,’ and that ‘*… reproductive trials in the rat, rabbit and monkey showed the antibiotic to be non-teratogenic*’.[86]

### 3.3. Potential for adverse dental effects

In the early 1960s, clinical evidence on tetracycline-associated tooth discoloration started to emerge.[6,46,87,88] It was soon established that tetracycline chelates calcium orthophosphate to form a complex that is irreversibly incorporated into teeth during the calcification stage of tooth development.[47,70,89] This resulted in permanent discoloration of the affected adult denture, because remodeling and calcium exchange do not occur after calcification is complete. A reduction of tetracycline tooth-staining incidence was achieved by dose reduction and avoiding its use during the critical period of tooth mineralization.[48,61]

*In utero* tooth formation begins in the sixth week of development, with tooth calcification setting in around the twelfth week of fetal life for deciduous teeth and at 3–4 months post-birth for permanent teeth.[90–93] Tetracycline exposure *in utero* leads to permanent discoloration of deciduous teeth only – upon exfoliation of these, the condition is completely resolved. Staining is of cosmetic significance only with no confirmed linkage to enamel hypoplasia. Tooth discoloration is the main adverse effect associated with tetracycline antibiotics in pregnancy, especially for exposure in the second or third trimesters.[3,6,9,46–49,61,70,94–98]

The overall incidence of tetracycline-associated tooth-staining during pregnancy is estimated at 3–6%.[99] The drug exposure time-point during gestation defines which teeth are stained.[50,100] However postnatal exposure to tetracycline from 3 months to 8 years of age results in life-long enamel discoloration of the primary and permanent dentition, in a dose and duration dependent manner.[47,51,52,61,89,94] After 8 years of age the tooth crowns are calcified and no permanent staining because of tetracyclines will occur.[47,52]

Oral tetracycline therapy has been associated with up to 40% restriction of bone growth, particularly of the fibula in preterm infants and in the second and third trimester of pregnancy. Since this growth retardation is reversible after discontinuation of treatment with rapid compensatory bone growth, this adverse effect has been regarded by some as a developmental delay and not a teratogenic effect.[61] There is no published evidence of any permanent structural skeletal defect in humans. In summary, tetracyclines are avoided during pregnancy because use after 25 weeks might result in staining of teeth in a small percentage of cases and possible effects on bone growth which are reversible.

Despite the lack of data on permanent human dental staining or decreased fetal bone growth associated with doxycycline use during pregnancy, the lingering fear of irreversible tooth discoloration remains (Table 2).[14,101] Doxycycline has a reduced ability to chelate calcium, and is less likely to cause permanent tooth discoloration than other drugs of its class.[46,87,98] The calcium-binding capacity is 19% for semi-synthetic doxycycline, which is two to threefold lower than the 39.5 and 74.5% for tetracycline hydrochlorate and dimethyl-chlor-tetracycline, respectively.[47,89,102] Lochary *et al*. highlighted the difficulties associated with longitudinal tooth discoloration studies and found no statistically significant difference in incidence or tooth-staining between doxycycline-treated cases (n = 10) and matched non-doxycycline exposed control subjects (n = 20).[53] Two recent reports confirmed similar observations in children exposed to doxycycline before the age of 8 years: The first study involved American–Indian reservation children who had received doxycycline treatment for suspected RMSF (n = 58, compared with 213 non-doxycycline exposed children) and were evaluated later upon eruption of permanent teeth – no tetracycline-like staining in any of the exposed children’s teeth was found (0/58, 95% CI 0–5%) and no significant differences in tooth shade (p* *= 0.20) or hypoplasia (p* *= 1.0) was found between the two groups.[103] The second blind, randomized, controlled clinical study examined children who had been treated with doxycycline in syrup form for controlling asthma attacks at a dose of 4 mg/kg bid on the first day followed by a single dose of 2 mg/kg/day for 9 days (n = 31, vs. 30 control non-doxycycline exposed asthmatic children). No tooth staining was detected in any of the children in either group.[104]

In animal studies, doxycycline usage was not linked to an increased incidence of skeletal anomalies until doses equivalent to 17 times the maximum human dose were used.[85] Although some women have been exposed to doxycycline in pregnancy, there is little human clinical data available for objective evaluation; the Hungarian Congenital Anomaly register identified 119 pregnancy exposures to doxycycline, predominantly in the first trimester, but teeth staining or bone growth problems were not assessed in these children.[64] Ten years later, a publication based on the same register reported 78 births; with 47, 20 and 11 cases that reported oral doxycycline treatment (200 mg on day 1, and 100 mg daily for 6 days i.e. short-term exposure) in the 1^st^, 2^nd^ and 3^rd^ trimesters respectively. Treatment during the first and second trimesters of pregnancy resulted in longer gestational age at delivery and a reduction in the rate of preterm births (based on three births); and mild intrauterine fetal growth retardation in five births between the thirty-first and thirty-fourth gestational week, however this did not contraindicate doxycycline use.[105] Since the reduced use of doxycycline is a consequence of the ‘tetracycline class effect’ based on fear of, but no evidence for tooth-staining and bone growth impediment, re-consideration of adequate clinical evaluation of doxycycline seems sensible. Although the US FDA states that the risk of an association with tooth staining cannot be eliminated due to the insufficient availability of supporting data, it is likely to be minimal.[106]

Recently, sporadic cases of a reversible and superficial discoloration of permanent teeth in adults undergoing doxycycline or minocycline treatment were associated with degradation products in the saliva, poor dental hygiene and possible UV exposure. This discoloration is not based on drug-complex incorporation, and can easily be removed using abrasive measures.[54,107,108]

### 3.4. Potential for adverse hepatic effects

Despite the well-documented association between tetracycline and liver toxicity, there is no evidence that links doxycycline to hepatotoxicity. In the early 1960s a series of fatal hepatotoxicity cases were linked to intravenous tetracycline therapy, when i.v. dosage recommendations reached up to 6 g/day.[2,4,5,7,8,10] Contributing factors were high doses applied over prolonged periods, often with co-existing risk factors like renal impairment (>60% of tetracycline is renally excreted) or in combination with other hepatotoxic agents (chloramphenicol or sulpha drugs).[2,4,10]

A large population-based case–control study compared exposure to doxycycline or tetracycline in patients with a diagnosis of hepatotoxicity using California Medicaid claims.[67] Of 22,605 individuals with a diagnosis of hepatotoxicity, 3377 (14.9%) participants were exposed to either tetracycline or doxycycline. Current and past users of tetracycline had a significantly increased risk of developing hepatotoxicity compared to age-gender matched healthy controls [OR (95%CI); current users 3.70 (1.19–11.45); past users 2.72 (1.26–5.85)], while doxycycline users did not [current users 1.49 (0.61–3.62); past users 1.74 (0.99–3.06)]. As doxycycline showed no increased risk of hepatotoxicity when compared to controls, the risk of liver damage was considered negligible, and the authors concluded that: *‘Doxycycline could potentially be a safe substitute for tetracycline, when appropriate’*.[67,72,109] The British National Formulary states that doxycycline is excreted primarily by the gastrointestinal tract and considered safer to use in patients with pre-existing renal disease than other tetracyclines with regards to hepatotoxicity.[110–113]

## 4. Discussion

### 4.1. Doxycycline is an important drug

Historically, the tetracyclines represent an important class of antibiotics, which have largely been replaced by much better tolerated and more effective semi-synthetic derivatives such as doxycycline. Unfortunately, inadequate data and limited evidence regarding the safety and efficacy of drugs during pregnancy have led to fear of teratogenicity and drug toxicity, and has embedded possible unjustified concerns regarding adverse fetal, neonatal or pediatric consequences. If this has resulted in the use of inferior therapies for the management of uncomplicated infections remains to be investigated, but is probable.[14]

Doxycycline is useful as an empirical treatment because of its high cost–effectiveness and wide spectrum of activity.[114–121] Large prospective studies addressing the causes of undifferentiated febrile illnesses have shown that up to 39% of patients could potentially benefit from treatment with doxycycline.[122–124] The major doxycycline-treatable rickettsial illnesses – scrub typhus, murine typhus and leptospirosis – represent the leading treatable etiologies of febrile illness in many regions across Asia. They are responsible for substantial morbidity, mortality and can severely affect pregnancy, notably worse than malaria, with high abortion rates and adverse neonatal outcomes.[123,125–130] Countries with a high estimated burden of these illnesses include India, Bangladesh, Myanmar, Laos, N-Vietnam, Korea and China, where currently doxycycline is not routinely prescribed for the empirical treatment of undifferentiated febrile illnesses. The increasing number of reports on rickettsial illnesses and leptospirosis emerging from these countries illustrate the potential impact an affordable and effective drug like doxycycline could make.

### 4.2. Doxycycline and the new FDA drug labeling requirements

Current doxycycline dosages are more than 10-times smaller than the analogous tetracycline dosages of nearly 50 years ago and treatment durations are shorter than 14 days.[15,30,33,39,40,110,131,132] However, the unequivocal benefits of doxycycline remain overshadowed by its tetracycline derived US FDA Class D classification. The risk of misclassification on the basis of ‘Class Effects’ was stated over 10 years ago by the FDA [133]: *‘Understanding the structure/activity relationships and pharmacological mode of action of a class of therapeutic agents in some circumstances can provide a prediction of the possible safety and efficacy of a new agent. However, such knowledge is generally not predictive of human teratogenesis*.[134] *For example, thalidomide and glutethimide are closely related by chemical structure, but there is no evidence that glutethimide is teratogenic*.[135]*’*

In June 2015, the FDA replaced the ‘Category classification for drug use in pregnancy’ with an evidence-based system using available information about each drug.[136] With this non-binding communication the prescribing decisions during pregnancy and lactation can now be reviewed with individualized and improved maternal, fetal and infant risk–benefit considerations.[136,137] This new FDA Drug Labelling Requirements and the move away from pregnancy categories could mean a renaissance for doxycycline and offers the possibility to rebuild its reputation. Small steps in this direction had previously been made, in that doxycycline treatment in pregnancy was accepted in life-threatening situations such as exposure to bio-threat agents.[13] Similarly, the Committee on Infectious Diseases of The American Academy of Pediatrics had revised the RMSF treatment options to include the use of doxycycline for young children.[53,138]

### 4.3. Doxycycline is under appreciated and under studied

This review collated the evidence that: (i) no teratogenicity during pregnancy; (ii) no permanent tooth-staining in pregnancy and in children under 8 years old; (iii) no hepatotoxicity and (iv) no permanent inhibitory bone growth effects (those of tetracycline are completely reversible), occur because of doxycycline use.

To date there are no controlled studies addressing SAEs of doxycycline in human pregnancy – a concerning and important symptom of the actual problem. The evidence shows that the risk of doxycycline-associated hepatotoxicity, teeth and bone-associated adverse effects and teratogenicity are negligible, but that good quality data is lacking.[14,63,64,79,83]

The absence of SAEs in the literature of doxycycline does not mean that these do not occur; it is possible that SAEs occur, but at rates that compare to controls – doxycycline can be considered safe in the first trimester of pregnancy and in children. If the rare and non-severe side effects of doxycycline are outweighed by the infection-associated risks, complications or death for mother and/or child, then its use should be considered.

These data, and the emerging knowledge on the worldwide impact of doxycycline-treatable febrile illnesses, strongly suggest that increased research efforts should be directed towards evaluating doxycycline in clinical treatment trials and in dosage-optimization pharmacokinetic studies, especially in pregnant women and children.

### 4.4. Poor perception of doxycycline’s potential

In 2009, the US FDA stated that while the risks of AEs and SAEs were minimal, the risk of doxycycline-associated tooth-staining could not be disregarded and required more robust AE data than currently available.[106] The FDA have also stated that the risk of doxycycline during pregnancy is likely to be minimal and others have advocated its use in malaria considering the very poor outcome seen with malaria in pregnancy.[106] Notably, emerging data suggests that rickettsial illnesses compare to malaria in terms of poor neonatal outcome.[125] The current situation with the treatment of rickettsial infection in pregnant women is similar to the situation with malaria in pregnancy a decade ago. Historically, the harmful effects of malaria in pregnancy continued unabated while quinine (considered safe in pregnancy) was used to treat pregnant women many years after its use in non-pregnant patients ceased because of the development of more efficacious and better-tolerated alternatives. Pregnancy exposure registries are useful to monitor drug safety in pregnancy and can provide information on the potential risk associated with certain drugs. However, antimalarial pregnancy registries yielded very poor results because of the difficulties of data linkage. In the case of rickettsial infections an effective drug is available – doxycycline, the drug of choice – but its use in pregnancy is prevented by its class assignment. Increased acceptance of the use of doxycycline in pregnancy has the potential to significantly reduce the mortality and morbidity from rickettsial infections in pregnancy.[125]

### 4.5. Doxycycline – are there alternative drugs?

Alternative drugs such as azithromycin and possibly clindamycin require further investigation, although these drugs are five times (clindamycin) and 20 times (azithromycin) more expensive than doxycycline (5-day adult treatment course, Thailand, 2015). Data on azithromycin in treating tropical rickettsial illnesses and leptospirosis is emerging, but not for clindamycin.[123,124] More evidence in pregnancy is required on safety, efficacy, dosage regimens and their potential role in salvage regimens for doxycycline treatment failures.[139] It is alarming that no studies are available on pharmacological dosage-optimization for these important drugs, or confirm if the currently recommended dosages lead to therapeutic plasma levels in pregnancy, early childhood, and for nasogastric tube or intravenous drug administration.

Efforts to conduct pharmacovigilance for tropical infectious diseases in pregnancy in low resource settings have failed.[140] Most developing countries rely heavily on safety data collated in industrial countries but drugs such as artemisinin derivatives used to treat malaria (embryotoxic at low dose ranges) are not widely used in countries with automated databases. Chronic diseases form the bulk of current pregnancy exposure registries, with transient infections being more difficult to capture in terms of record linkage, accurate determination of gestational age and confirmation of newborn examination.[140]

### 4.6. Study limitations

There are several limitations to this study. Study selection bias is likely because of the limitations of electronic searches of old literature relating to the pre-digital era of tetracycline discovery and the use of reference lists to identify articles, the exclusion of unobtainable studies, and a pronounced under representation of modern and systematic studies. Further, doxycycline-associated studies related to dental procedures, skin conditions (mainly acne), infertility, STI, genitourinary infections, cancer or ill-defined endpoints (i.e. tooth staining) had to be excluded, resulting in limited data for pregnant or pediatric patients. These limitations however have been addressed in detailed discussions in the text, as some limitations themselves represent a consequence of the neglect this drug has received in the past.

## 5. Conclusion

This systematic review of the available literature on doxycycline use in pregnant women and pediatric populations has identified that restrictions for the use of doxycycline in human clinical trials exist, but that pursuing prospective re-evaluation of doxycycline is indicated. Although doxycycline is a semi-synthetic derivative of tetracycline, the available (albeit limited) evidence disassociates it from the permanent tooth discoloration, bone growth disturbance and teratogenicity seen with tetracycline when administered during pregnancy or early childhood. The change of the labeling requirements by the FDA has greatly facilitated the procurement of better data on the use of doxycycline in common tropical illnesses and in vulnerable populations. The shift from a categorical system to an improved risk–benefit assessment for drugs used during pregnancy and lactation will enable clinical researchers to properly evaluate the true safety and efficacy profile of doxycycline. The data presented in this review argues for a ‘*softening of restrictions*’ on the use of doxycycline in the first trimester/half of pregnancy, as well as better advocacy for its use in early childhood. Alternative drugs like azithromycin should be evaluated in parallel for the treatment of common causes of undifferentiated fever. Pharmacological studies of doxycycline and azithromycin in pregnant women and children would inform dosage-optimization and contribute to improving regional/national empirical treatment guidelines.

## 6. Expert opinion

### 6.1. What are the key findings and weaknesses in the research done in this field so far?

Doxycycline is inexpensive and highly effective in treating infections of high global impact including malaria (prevention and as a partner drug for treatment), sexually transmitted infections (pelvic inflammatory disease, chlamydia, syphilis), rickettsial illnesses (leading causes of treatable undifferentiated febrile illness in Asia), Lyme disease, skin infections and acne. Doxycycline is useful as an empirical treatment for suspected leptospirosis and rickettsial infections due to its high cost–effectiveness, wide spectrum of activity and bioavailability.

Unfortunately, the widespread belief of many clinicians is that doxycycline is contraindicated in pregnant and pediatric patients because of the risks of permanent mother or child tooth discoloration, teratogenicity and hepatotoxicity. However, the classification of doxycycline is based on inadequate data and limited evidence regarding the safety and efficacy in these patient groups, which consequently has led to insufficient clinical and pharmacological evaluation in pregnant women and children. Over time concerns regarding adverse fetal, neonatal or pediatric consequences, have become embedded, despite being unjustified by the evidence. The available literature supports a safety profile that differs significantly from tetracycline, and no correlation between the use of doxycycline and teratogenic effects or dental staining can be found.

### 6.2. What potential does this research hold? What is the ultimate goal in this field?

Evaluating the clinical treatment efficacy of doxycycline and the more expensive alternative drug azithromycin in pregnancy and early childhood could lead to a renaissance for these antimicrobials. Emerging data on the high prevalence of doxycycline-responsive illnesses in resource-poor regions, their associated morbidity and case-fatality rates and the devastating impact of easily-treatable rickettsial illnesses in pregnancy and CNS infections are strong arguments to revisit and reconsider the indications for and use of doxycycline. Doxycycline has the potential to make a significant impact on perinatal morbidity and mortality, especially in low to middle income countries.

### 6.3. What research or knowledge is needed to achieve this goal and what is the biggest challenge in this goal being achieved?

There is a complete lack of pharmacometric studies for doxycycline, and very limited data for its best alternative; azithromycin, especially in febrile pregnant women, children and patients with severe rickettsial or leptospiral infections. No guidance for optimal dosing is available, and no alternative treatment options or pregnancy-compatible combination therapies including single dose or short courses of doxycycline with azithromycin have been evaluated.

Recent longitudinal studies in children treated for Rocky Mountain Spotted Fever (RMSF) have shown that doxycycline in the currently recommended dosage schedule is not associated with dental discoloration. Early initiation of effective empirical treatment, to bypass the diagnostic difficulties associated with rickettsial infections, can save lives and prevent severe disease. The biggest challenge in achieving a broader evaluation of this antimicrobial is to change the perception of adverse effects in health care professionals – with the non-binding communication recently released by the FDA, the prescribing decisions during pregnancy and lactation have now been shifted towards a more evidence-based approach. This represents a significant improvement over the previous pregnancy category classification, and for doxycycline the new FDA Drug Labelling Requirements now enable and support the procurement of evidence to rebuild its reputation.

### 6.4. Where do you see the field going in the coming years? What is going to happen?

Increasing appreciation of the significant impact of doxycycline-treatable diseases will become apparent in the coming years. Large prospective multicenter studies have confirmed the treatable causes of febrile illness in many tropical regions. For example in Laos an estimated 39% of patients presenting with an acute febrile illness would potentially benefit from treatment with doxycycline. Complications with substantial mortality, and severe disease with CNS infections are reported from countries across Asia, representative of the large treatable and preventable disease burden in countries like India, Bangladesh, Myanmar, Laos, N-Vietnam, Korea and China, where doxycycline is currently not routinely prescribed for empirical treatment. These emerging data illustrate the potential importance of a highly effective and affordable drug like doxycycline in populous regions across Asia. It can be anticipated that as more safety, efficacy and pharmacometric data become available, this antimicrobial will be used increasingly, not only for directed treatment, but also as potential empirical treatment strategy in resource-poor settings where diagnostics are limited. Ideally this would be conducted alongside pregnancy pharmacovigilance.

### 6.5. Is there any particular area of the research you are finding of interest at present?

Areas of research of particular interest at present include clinical studies focusing on the identification of the causes of febrile illness and serious complications. The emerging data from these studies will lead to re-evaluation of national guidelines, inform treatment strategies and emphasize the need for easily available and affordable diagnostics.

Other exciting areas of research include the development, evaluation and validation of bedside diagnostics and the conduct of pharmacological studies in vulnerable populations such as pregnant women, children and patients with severe infections requiring nasogastric tube feeding and intravenous antimicrobials.

## Supplementary Material

supplementary_files.zip

## Article highlights

- Doxycycline is avoided during pregnancy because other tetracyclines have been associated with transient suppression of bone growth and with staining of developing teeth.
- Doxycycline is not dangerous and its classification misleading; this systematic review found evidence for the absence of: (i) teratogenicity during pregnancy; (ii) permanent tooth-staining in pregnancy and in children under 8 years old; (iii) hepatotoxicity and (iv) permanent inhibitory bone growth effects.
- Doxycycline is safe in early pregnancy, possibly throughout pregnancy and for children at the current dosage regimes; the restraints on doxycycline could be lifted for targeted or empiric treatment during the first half of pregnancy and for children under 8 years of age, if the daily dosage does not exceed 200mg/day for a maximum duration of 14 days.
- The poor neonatal outcome of rickettsial illnesses in pregnancy represents a high fetal risk, notably higher than in malaria, which vastly outweighs the risks and consequences of doxycycline treatment side effects.**Research gaps**
- Clinical treatment efficacy trials for doxycycline and alternative drugs like azithromycin are urgently required, especially in pregnancy and early childhood.
- Pharmacokinetic studies for these drugs should be prioritized, especially with a focus on pregnant women, children and rickettsial infection patients.
- Treatment options for short courses and pregnancy-compatible combination therapies including single dose or 3-day courses of doxycycline with azithromycin should be evaluated.
- Longitudinal studies addressing dental discoloration and bone growth issues associated with doxycycline should be pursued (does a 7–14 day course of doxycycline have any visible longitudinal effects on teeth at a later stage of life?).

This box summarizes key points contained in the article.
