# Supplementary material for: Revisiting doxycycline in pregnancy and early childhood – time to rebuild its reputation?
Source: Expert Opin Drug Saf. 2016 Jan 25;15(3):367–82. doi: 10.1517/14740338.2016.1133584 (PMC4898140; doi:10.1517/14740338.2016.1133584)
Supplement: supplementary_files.zip [file ieds_a_1133584_sm9366.zip › supplementary files/S2_Doxy_PRISMA_protocol_R1.docx]

**Systematic Review Protocol**

**Review title:**

Revisiting doxycycline in pregnancy and early childhood – time to rebuild its reputation?

**Reviewer roles**

Primary reviewers: Ruby Cross^1^ and Clare Ling^1,2^

Secondary reviewers: Rose McGready^1,2,3^ and Daniel H. Paris^2,3^

Independent reviewer: Nicholas P.J. Day^2,3^

Quality assessors: Rose McGready^1,2,3^, Nicholas P.J. Day^2,3^ and Daniel H. Paris^2,3^

^1^Shoklo Malaria Research Unit (SMRU), 68/30 Ban Toong Road, Mae Sot, Tak 63110, Thailand.

^2^Mahidol-Oxford Tropical Medicine Research Unit (MORU), Faculty of Tropical Medicine, Mahidol University, 420/6 Rajvithee Road, 10400 Bangkok, Thailand.

^3^Centre for Tropical Medicine & Global Health, Nuffield Department of Clinical Medicine, University of Oxford, Old Road Campus, Roosevelt Drive, Oxford, OX3 7FZ, UK

**Center conducting the review**

Shoklo Malaria Research Unit (SMRU) and Mahidol-Oxford Tropical Medicine Research Unit (MORU)

Faculty of Tropical Medicine, Mahidol University, 420/6 Rajvithee Road, 10400 Bangkok, Thailand

**Study rationale**

The overall aim of the project is to raise the profile of doxycycline, and create awareness that it is required to treat a huge patient population at risk for treatable undifferentiated fever, and that doxycycline is not as dangerous as perceived by doctors and clinical researchers alike. In this review, we aim to reveal the exact underlying data – what is known and identify what we need to know – to improve the (empiric) treatment of undifferentiated febrile illnesses. In December 2014, the FDA announced that it is going to drop the Category classification for drug use in pregnancy, and replace it with a system giving information on what knowledge there is about each drug. We aim to prepare the data in view of this announcement to provide arguments for “loosening” the restrictions on doxycycline, which should promote and facilitate pharmacological investigations, both PK/PD and drug efficacy studies, of this drug – especially in pregnancy and young children/infants.

**Review questions/objectives**

The quantitative objective/s is/are to identify

“What are the justifications of the current restrictions on the use of doxycycline in pregnant women and early childhood?”

More specifically, the objectives are to identify:

1. To critically summarise the published literature on the therapeutic benefits/limitations of doxycycline

2. To collate the evidence that is restricting its use in human clinical trials in pregnancy and childhood

- Focus on: teratology effects / teeth staining / bone growth / hepatotoxicity and nephrotoxicity

3. To identification relevant research gaps.

**Background**

Doxycycline is a very effective antimicrobial drug for the treatment of various infectious diseases, including Lyme disease, anthrax and rickettsial illnesses. It has a broad therapeutic spectrum and exceptional bioavailability, but it is a tetracycline analogue and as such classified by the FDA as a class D drug, contraindicated in pregnancy and for children less than 8 years of age.

We aim to review the evidence on doxycycline use in pregnant women and pediatric populations and identify major research gaps.

Given the high incidence and consequences of doxycycline-treatable diseases including rickettsial and leptospiral infections in some areas such as Southeast Asia, and the overall benign side effect profile, the current restrictions on its use in pregnant women and early childhood should be re-considered.

Critical review of evidence should include understanding the current restrictions by the FDA and reveal important clinical research gaps.

We would like to perform prospective evaluations of alternative antimicrobials and clinical treatment trials that should include doxycycline. Further, more in-depth studies on doxycycline pharmacokinetics supporting dosage-optimization and modelling in pregnant women and children would impact disease outcomes in these vulnerable populations.

**Inclusion criteria**

All studies relating to the use of doxycycline and pregnancy and children, with a focus on side-effects, including hepatotoxicity or teeth or teratogenicity in the fetus or infant.

**Outcomes and intervention/phenomena of interest**

The primary outcomes of the analysis will relate to all relevant severe adverse effects for doxycycline in literature and include irreversible tooth-staining; bone growth inhibition; teratogenicity.

The secondary outcome measures will include hepatotoxicity and nephrotoxicity (and other relevant side effects) relating to doxycycline.

For comparisons, this requires insight into tetracycline adverse effects, but should not lead to a complete tetracycline review, as this data has been corroborated sufficiently (see Reprotox databases).

**Types of studies**

This review will consider for inclusion all types of experimental and epidemiological study designs, including but not limited to randomized and non-randomized controlled trials, quasi-experimental before and after studies, prospective and retrospective cohort studies, case control studies and analytical cross sectional studies, descriptive epidemiological studies, as well as opinion papers and reports.

Issues related to cost effectiveness, cost benefit, cost minimization, cost utility will not be addressed, as this is the focus of a separate project.

**Search strategy**

The search strategy aims to find both published and unpublished studies, using following approach:

Articles will be searched for using electronic resources, through scanning of library index catalogues and reference lists of relevant articles.

This is followed by an electronic search using the PubMed electronic database using following terms: “doxycycline” and “hepatotoxicity OR pregnancy OR teeth OR side-effects”, and results reviewed manually.

Thirdly, the reference list of all identified reports and articles will be searched for additional studies. Studies published in English language will be considered for inclusion in this review. No date limitations exist for inclusion/exclusion in this review.

Subsequent cleaning of the database for errors, duplications, themes that do not relate to the review objectives (p.ex. dental or skin or cancer issues or other animal or lab-based studies not related to the objectives of the study) – and create a flow chart delineating this.

**Assessment of methodological quality**

All reports/papers selected for retrieval will be assessed by two independent reviewers for methodological validity prior to inclusion in the review. Any disagreements that arise between the reviewers will be resolved through discussion with the secondary reviewers, or with the third independent assessor.

**Data collection**

Quantitative and qualitative data on adverse effects of the drug and arguments against the use of doxycycline will be extracted from papers and included in the review in form of a comprehensive table (either incorporated into manuscript or as an appendix). The extracted data will include specific details about the interventions, populations, study methods and outcomes of significance to the review objectives.

**Data synthesis, discussion and Context**

Results will be pooled into a summary table with the major associations found contributing to the current restrictions on the use of doxycycline in pregnant women and early childhood.

Available data amenable to statistical meta-analysis will be included and analyzed. We anticipate that the lack of data and difficulties with the objectivity of endpoints (teeth staining) will be limiters for the statistical pooling of results.

Effect sizes expressed as odds ratio (for categorical data) and weighted mean differences (for continuous data) and their 95% confidence intervals will be calculated for analysis. Heterogeneity will be assessed statistically using the standard Chi-square and also explored using subgroup analyses based on the different quantitative study designs included in this review. If statistical pooling is not possible, the findings will be presented in narrative form including tables and figures to aid in data presentation where appropriate.

**Authors of the protocol**

v1 Daniel H. Paris (Jan 2015)

v2 Rose McGready, Nicholas P.J. Day, Daniel H. Paris (Feb 2015)

**Appendix 1:** **Data extraction criteria (for selected pdfs)**

| **Criterion** | **To describe / identify** | **Details** |
| --- | --- | --- |
| Study method? | [RCT, retrospective, observational, longitudinal, other]  [describe in words] |  |
| Sample size? | [number, include subgroups, controls]  subgroups? |  |
| Intervention? | Approach – congruent to other studies to allow metanalysis (data pooling)?  [describe in words] |  |
| Study Endpoints and/or Outcomes? | Are they congruent with our study question? [describe outcomes] |  |
| Statistical methodologies valid? | Outcomes and statistical calculations valid? [list OR, RR, p-values etc.] |  |
| Inclusion criteria detailed? | important for comparisons / data pooling [describe in words] |  |
| Ethically sound? | Approval / reviews detailed?  [human clinical studies only] |  |
| Conclusions drawn? | [describe – are they valid?] |  |
| Possible bias of the study? | [inclusion/exclusion criteria, endpoints, interpretation of results, selection bias, recall or information bias etc. | [over/under estimation?] |
| Results generalizable? | Extrapolation of results to population possible? |  |
| Summary – Evidence quality | Evaluate the quality of evidence (GRADE score) |  |
| **General Comments** |  |  |
| Name, date and signature |  |  |

Criteria were selected from appraisal checklists, based on the Joanna Briggs Institute (JBI) Qualitative and Assessment and Review Instrument (QARI) Data Extraction Form for Interpretative & Critical Research

**GRADE score**:

high – more research is unlikely to change our confidence

moderate – more research is likely to have an important impact on confidence and may change the estimate

low – more research is very likely to have an important impact on confidence and is likely to change the estimate

very low – we are uncertain about the estimate
